# Comparison of chromosomal instability of human amniocytes in primary and long-term cultures in AmnioMAX II and DMEM media: A cross-sectional study

**DOI:** 10.18502/ijrm.v13i10.7773

**Published:** 2020-10-13

**Authors:** Seyed Mehdi Hoseini, Fateme Montazeri, Maryam Moghaddam-Matin, Ahmad Reza Bahrami, Hassan Heidarian Meimandi, Saeed Ghasemi-Esmailabad, Seyed Mehdi Kalantar

**Affiliations:** ^1^Department of Biology, Faculty of Science, Ferdowsi University of Mashhad, Mashhad, Iran.; ^2^Biotechnology Research Center, International Campus, Shahid Sadoughi University of Medical Sciences and Health Services, Yazd, Iran.; ^3^Abortion Research Center, Yazd Reproductive Sciences Institute, Shahid Sadoughi University of Medical Sciences, Yazd, Iran.; ^4^Research and Clinical Center for Infertility, Yazd Reproductive Sciences Institute, Shahid Sadoughi University of Medical Sciences, Yazd, Iran.

**Keywords:** Human amniotic fluid cells, Chromosomal instability, Pseudomosaicism, Amniocentesis, Replicative senescence.

## Abstract

**Background:**

The genomic stability of stem cells to be used in cell therapy and other clinical applications is absolutely critical. In this regard, the relationship between in vitro expansion and the chromosomal instability (CIN), especially in human amniotic fluid cells (hAFCs) has not yet been completely elucidated.

**Objective:**

To investigate the CIN of hAFCs in primary and long-term cultures and two different culture mediums.

**Materials and Methods:**

After completing prenatal genetic diagnoses (PND) using karyotype technique and chromosomal analysis, a total of 15 samples of hAFCs from 650 samples were randomly selected and cultured in two different mediums as AmnioMAX II and DMEM. Then, proliferative cells were fixed on the slide to be used in standard chromosome G-banding analysis. Also, the senescent cells were screened for aneuploidy considering 8 chromosomes by FISH technique using two probe sets including PID I (X-13-18-21) & PID II (Y-15-16-22).

**Results:**

Karyotype and interphase fluorescence in situ hybridization (iFISH) results from 650 patients who were referred for prenatal genetic diagnosis showed that only 6 out of them had culture- derived CIN as polyploidy, including mosaic diploid-triploid and diploid-tetraploid. Moreover, the investigation of aneuploidies in senesced hAFCs demonstrated the rate of total chromosomal abnormalities as 4.3% and 9.9% in AmnioMAX- and DMEM-cultured hAFCs, respectively.

**Conclusion:**

hAFCs showed a low rate of CIN in two AmnioMAX II and DMEM mediums and also in the proliferative and senescent phases. Therefore, they could be considered as an attractive stem cell source with therapeutic potential in regenerative medicine.

## 1. Introduction

Since 1990s, when studies revealed the proliferation and differentiation potentials of amniotic fluid cells (AFCs), they have been considered as an attractive source for stem cells. In the last decade, amniotic fluid-derived stem cells have been particularly considered for cell therapy and regenerative medicine (1-4). The AFCs are a heterogeneous population of various fetal cell types (5, 6) particularly, mesenchymal stem cells (MSCs) (7). Amniotic fluid-derived MSCs have some advantages over MSCs derived from adult sources, for instance, they show higher clonogenicity, faster growth in differentiation capacity and privileged plasticity (8). Despite the potential therapeutic use in clinical procedures, MSCs have a weak point in in vitro conditions. Generally, one of the main drawbacks in culture and ex vivo expansion of stem cells including MSCs is a phenomenon called chromosomal instability (CIN) in which the loss and gain of some or whole chromosomes establishes aneuploidy and polyploidy in cultured cells, respectively (4).

Although the genetic stability of stem cells to be used in cell therapy and other clinical applications is strongly critical, the relationship between in vitro expansion of stem cell lines and their genomic stability has not yet been completely elucidated. The literature shows that there is no agreement between authors about the existence and frequency of CIN among different sources of stem cells including adult stem cells like mesenchymal stem/stromal cells (MSCs) (9), neural stem cells (NSCs) (10), and pluripotent stem cells (11). Although, many studies have well indicated the high rate of autosome and sex chromosomal abnormalities in human MSCs (12), on the contrary, further studies described chromosomal stability in culture for MSCs from different tissue sources (13). Depending on the origin of stem cells, culture conditions, incubation times and study design, the different levels of CIN are reported in the primary or long-term cultures (12).

In recent years, derivation of stem cells from human fetal sources such as placenta, amniotic fluid, amnion, and umbilical cord blood has introduced a fascinating alternative for stem cell therapy. However, these sources need to be investigated for chromosomal stability in culture conditions because increasing evidence has revealed that the therapeutic use of MSCs derived from different sources could be troubled by a time-dependent accumulation of chromosomal abnormalities and subsequently malignant transformation during ex vivo expansion. On the contrary, a literature review reveals that there are few studies pertaining to the culture method-related chromosomal stability in fetal stem cells. The observed low rate of CIN in the early cultures of human-AFCs by both karyotype and interphase fluorescence in situ hybridization (iFISH) methods motivated us to investigate the effect of different culture medium and long-term cultures on the chromosomal status of these cells.

## 2. Materials and Methods

### Sampling and experimental design

In this cross-sectional study, 15 sample of Human-AFCs from 650 samples were taken from high-risk pregnant women who were referred for diagnostic procedure of amniocentesis to the Yazd Reproductive Sciences Institute, cytogenetic laboratory, prenatal diagnosis (PND) section, between Oct. 2014 to Sep. 2018. Approximately, 15-20 ml human amniotic fluid was collected under the guidance of ultrasonography from pregnant women at second trimester, ranging from 16^th^ to 19^th^ weeks of pregnancy. Of note, the part of our information regarding the effect of primary cultures on CIN in these cells was obtained from patient's records. At the beginning, for fast prenatal genetic diagnosis, a fraction of 2.5 ml of amniotic fluid was used for simultaneous detection of aneuploidy in chromosomes 13, 18, 21, X, and Y in uncultured hAFCs by interphase fluorescence in situ hybridization (iFISH) technique. Considering that aneuploidies in these five chromosomes are able to reach the term, their early diagnosis for the termination of pregnancy is crucial. The remaining amniotic fluid were expanded in culture, usually for 2-3 passages, and examined for possible changes in the number and structure of whole chromosomes by G-banding karyotypes.

Aneuploidies detected in some cases need to be verified by uncultured results to make sure they really exist in fetus or are derived from culture condition. Accordingly, a comparison with uncultured results confirmed the fetal origination of all aneuploid cases which have been seen in karyotype of cultured amniocytes. After the clinical diagnosis, and regarding the chromosomal stability of hAFCs in primary cultures, we pursued to find: (1) the effect of culture conditions on chromosomal stability and (2) the study of chromosomal stability of hAFCs in long-term cultures. In order to achieve these goals, using a randomized cross-sectional study, we randomly cultured 15 samples of AFCs as long as they were able to proliferate. Moreover, each sample were cultured in two different culture mediums, DMEM and AmnioMAX II complete medium. In these cases, cells were passaged until they reached senescence, when no more growth was observed at least during one month. Finally, senescent AFCs were screened for aneuploidy and polyploidy regarding 8 chromosomes by FISH technique.

### Culture of hAFCs

After the clinical diagnosis and following 2-3 passages, 15 amniocytes samples were randomly collected for research purposes. At this time, cells were cultured in T25 flasks using two different mediums (1) Amniomax II with aforementioned supplements and (2) DMEM (Dulbecco's Modified Eagle Medium) supplemented with 4 mM L-Glu, 10 mM HEPES, 15% FBS (Fetal bovine Serum), and 1% PenStrep (all from Gibco Co. UK). In both groups, cells were incubated at 37°C under 5% humidified CO${}_{2}$. For the passage of the cells, usually at 80-90% confluency, amniocytes were treated with 1 ml of 0.05% Trypsin-EDTA (Ethylene Diamine Tetra Acetic Acid) for 1 min (Gibco Co. UK), deactivated by addition of the medium in two-fold volume and finally split at the ratio of 1 to 2.

### Karyotyping and FISH

During the clinical PND process, following the culture of AFCs, chromosomes were investigated for any numerical and structural abnormalities. Accordingly, amniocytes were harvested between passages 2-3 in a highly proliferative status by a fixative solution composed of acetic acid/methanol (1:3 v/v). Before fixation, cells were arrested by KaryoMAXⓇ Colcemid (Gibco Co. USA). Next, the fixed cells on the slides were treated by pancreatin (for chromosome banding) and then stained with Giemsa (Sigma-Aldrich Co. USA) to be used in standard chromosome G-banding analysis. Subsequently, according to the recommendations of the International System for Human Cytogenetic Nomenclature, cytogenetic analysis was performed depending on the karyotype of at least 25 metaphases (14).

Further, during the clinical fast diagnosis, the uncultured AFCs were investigated for aneuploidies with regards to 5 chromosomes including 18/X/Y and 13/21 using iFISH technique. Also, for research part of study, the FISH technique was applied to study the chromosomal stability of the cultured cells after senescence (passages 3-17) and under effect of two different mediums using eight probes for X-13/18/21 and Y-15/16/22 chromosomes. For this purpose, the harvested cells were fixed on the slides and then used for FISH pretreatment. The slides were incubated in a pepsin solution- 10 min for uncultured cells and 3 min for cultured cells (Leica Co. Amesterdam)- followed 3 min in 1x PBS, 10 min in 10% N-formaldehyde, and again washed for 3 min in 1x PBS to remove all unfixed cells and debris from the surface of the slides. For dehydration, the slides were placed in 70%, 85%, and 99.9% ethanol series each for 1 min. After the co-denaturation of the samples and probes at 75 °C for 5 min, locus-specific DNA multicolor probes (Metasystem Co. UK) were hybridized to chromosomes overnight. Analysis was performed using an Olympus BX61 semi-automized fluorescence microscope equipped with a triple-band pass filter for DAPI (diamidino phenylindole)/Texas Red/fluorescein isothiocyanate (FITC) and single-band pass filters for FITC, Texas Red, and Aqua blue. Two probe sets including PID I (X-13-18-21) and PID II (Y-15-16-22) from Metasystem Company (UK) were applied. Each sample was scored by two analysts, and the scoring was done strictly according to the standard criteria (15).

### Ethical consideration

All participants signed an informed consent form for the use of their amniotic fluid sample in the study and all records were required to be kept confidential. The study protocol was based on accordance with the guidelines of the ethical committee of the Shahid Sadoughi University of Medical Sciences and Health Services (code: IR.SSU.REC.1396.169).

### Statistical analysis

Data were categorical and presented in frequencies and percentages, when > 25% of the table cells had a frequency < 5%, Chi- square test was used for the statistical analysis. In tables with a smaller number of data frequency, Fisher's exact test with less power was used. Besides, comparisons of the chromosomal abnormalities rate between groups (under two different culture conditions) were performed by the Student's *t* test using the SPSS software (Statistical Package for the Social Sciences version 20.0, SPSS Inc., Chicago, IL, USA). P-value < 0.05 was considered as statistical significance.

## 3. Results

### CIN in uncultured hAFCs

Screening of aneuploidy for 5 chromosomes including 13, 18, 21, X, and Y by a fast iFISH-based technique was used for early PND. Given that only the uncultured cells or the cells from first or second passages were examined in this section, our finding showed a culture-derived chromosomal abnormality in these cells, which it could indicate the chromosomal stability of human AFCs in primary cultures (passages 2-3). It should be noted that the results of fast FISH-based technique were completely consistent with the karyotype results in early cultures until the third passage. Only one case of the mosaicism in sex chromosomes was observed due to the more number of cells that were analyzed in the fast FISH-based technique rather than karyotype. Of course, this case was excluded because of the detection of abnormality in < 10% of the cells (as errors caused by technical complications concerning FISH technique) (15).

### CIN in early-passaged hAFCs

**Figure 1:**
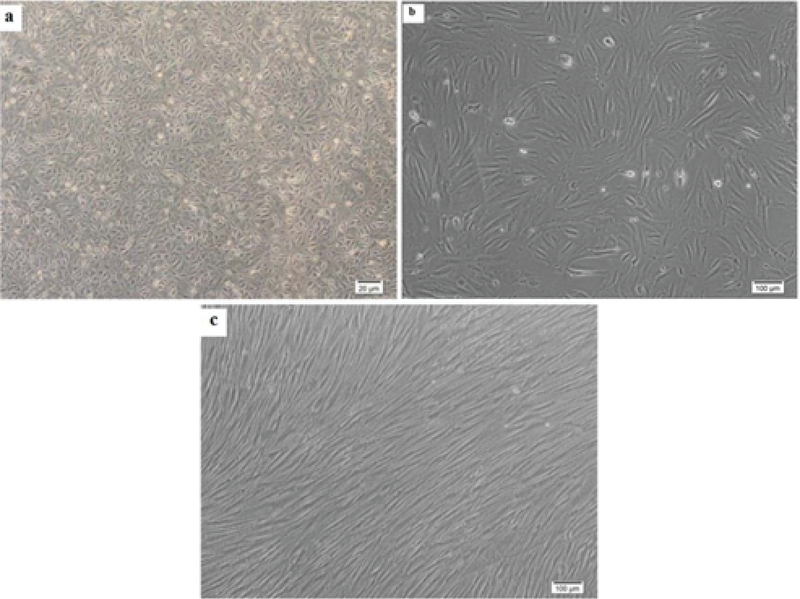
Different types of adherent amniocytes in long-term cultures. (a) Proliferating E-type cells with a columnar to cuboidal morphology at the 4th passage. (b) AF-type cells with a fibroblastic-like shape at 3rd passage. (c) Long spindle (LS) F-type cells in a highly proliferative status at the 8th passage.

**Figure 2:**
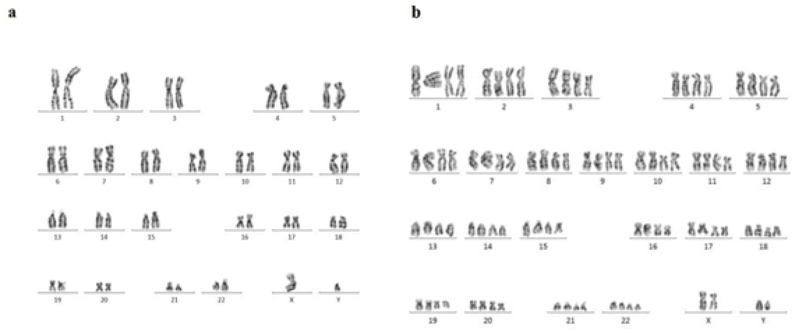
Karyotyping hAFCs. (a) Normal karyotype in hAFCs. (b) A tetraploid karyotype (92, XXYY) at the 3rd passage raised due to culture in a normal sample (46, XY).

In primary cultures, we need the adherent cells to be harvested in a proliferating status and use the metaphase for cytological analysis. Similar to previous studies, in our study, the hAFCs showed three entirely distinct morphologies (Figure 1). Because of their fetal origin, they all have the ability to proliferate and form colonies. Although there have been some abnormalities among the understudied cases, they all have originated from fetus. Among the detected abnormalities in this part, most frequency belonged to trisomy 21 and the lowest frequency for trisomy 13 (data not shown). Our findings indicated that none of the observed aneuploid cases raised from culture method; in other words, all aneuploidies in chromosomes 13, 18, 21, X, and Y were also verified by FISH analysis on uncultured amniocytes. The results achieved during the culture of amniocytes for maximum three passages showed that, among 650 cases, CIN was observed to be caused by culture only in 6 cases (< 1% of samples). The only CIN observed in these six cases was polyploidy including mosaic diploid-triploid and diploid-tetraploid (Figure 2). Therefore, based on our results, polyploidy in a few cells in < 1% of cases was the only CIN associated with culture method during the three passages of AFCs.

### CIN in long-term cultured human amniotic fluid cells under two culture conditions

**Figure 3:**
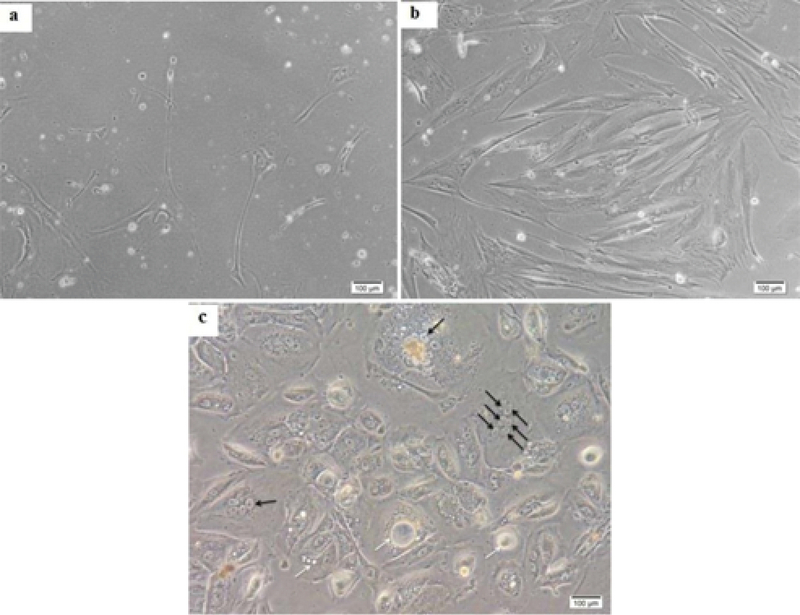
Different types of senescent cells, as diverse as their primary morphology. (a) Neuronal-like morphology with cytoplasmic projections in senescent fibroblastoid cells at the 17th passage. (b) AF-type cells at the beginning of senescence. (c) Flat, circular E-type cells at the 5th passage when they start being multinuclear (black arrows), granular, and vacuolar (white arrows).

In total, 15 amniocytes samples were randomly collected and cultured in two DMEM and AmnioMAX mediums until ceased to grow. Senescent AFCs enlarged and demonstrate an irregular morphology with cytoplasmic projections and multinuclear, granular, vacuolar appearance (Figure 3). Our finding showed a highly variable result, so that senescence could be observed during passages-17. In all samples, approximately 35-55 cells were analyzed for each panel of probes. Among the studied samples, one case was excluded from the study due to the lack of enough cells for FISH-based analysis. One out of fifteen understudied hAFCs showed fetal-origin trisomy 21, in which interestingly no further abnormality was established in culture following the six passages. Between 14 samples included in this study, maximum AFCs expansion was possible in up to 17 passages in AmnioMAX medium and 9 passages in DMEM, after which cells ceased to grow. Overall, our results showed that AFCs go through senescence significantly earlier when cultured in DMEM compared to the AmnioMAX medium (data not shown).

**Figure 4:**
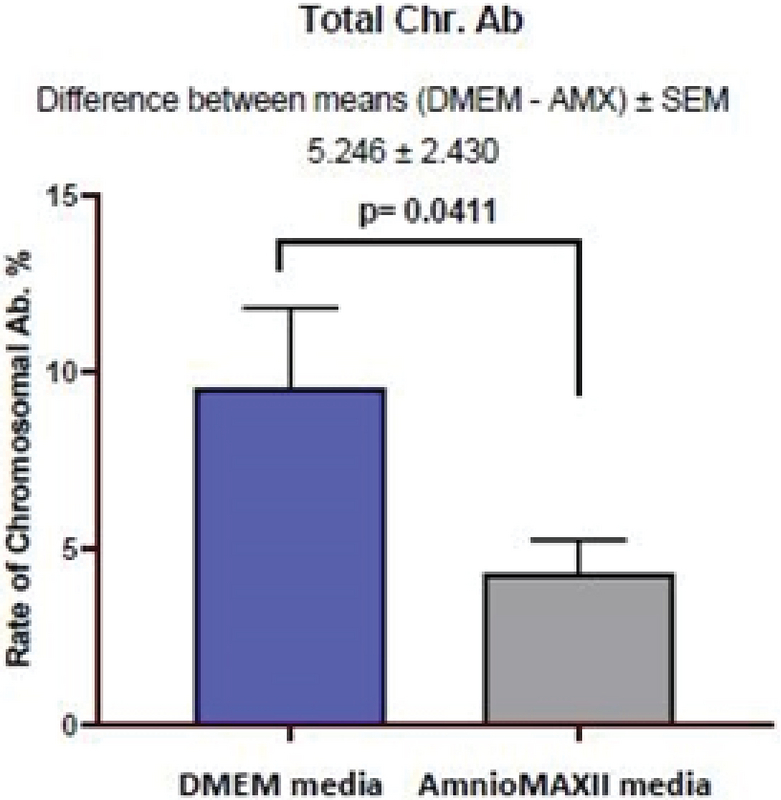
Comparison of the abnormalities in hAFCs under two different mediums (DMEM vs Amnio MAX II). Data are presented as Mean ± SEM. The information obtained from analyzing 535 cells under DMEM medium and 510 cells under Amnio MAX medium. Data are presented as Mean ± SD.

Moreover, investigating chromosomal abnormalities in senescent hAFCs, regarding the 8 chromosomes including X-13/18/21 and Y-15/16/22 by a FISH-based technique showed different aneuploidy and polyploidy (Figure 4). However, there have been normal samples without any types of culture-derived aneuploidy and polyploidy in both mediums, 4 and 3 out of 13 samples in AmnioMAX and DMEM groups, respectively. Furthermore, if we only consider aneuploidy, the number of normal cases would be even more, 8 and 6 out of 13 cases in the AmnioMAX and DMEM groups, respectively. Remarkably, taking chromosome 13 into account alone, all samples in AmnioMAX group were normal, though, differences between two groups regarding each investigated chromosome individually were insignificant (Tables I and II, p < 0.05). The most striking result to emerge from the data is the trivial frequency of aneuploidies regarding each chromosome, ≤ 1%. Even the mean of the total chromosomal abnormalities regarding all analyzed chromosomes was 4.16% in DMEM and 2.59% in AmnioMAX media, respectively. Considering the chaotic mosaicism, in which more than one chromosome is engaged in aneuploidy, the frequency of it was about 0.9% in DMEM-cultured cells and zero in AmnioMAX- cultured cells. Polyploidy was observed in cells from both culture mediums with frequencies around 5% and 1.7% in the DMEM and AmnioMAX, respectively.

**Figure 5:**
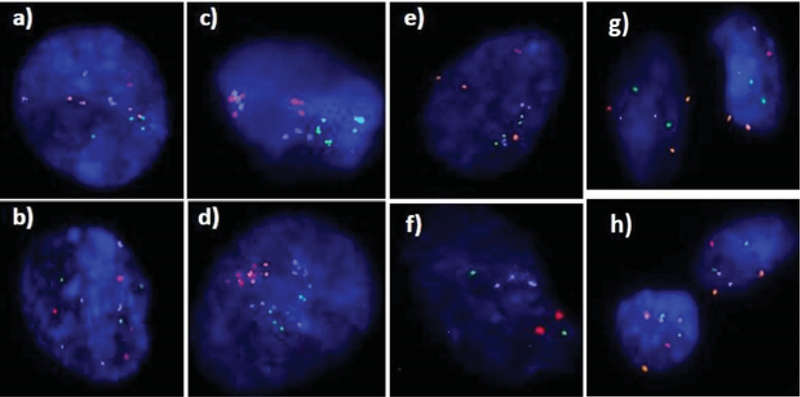
Abnormalities analyzed by staining 8 chromosomes using a FISH-based technique. Data are presented as Mean ± SEM. Figure show the example images of polyploidy, including tetraploidy for two probe sets (a & b), hexaploidy (c) and heptaploidy (d), and examples of chaotic include trisomy 22 and tetrasomy 15 and 16 in one cell (e) and nulisomy 21 and trisomy 18 in another cell (f). Images of g and h show an example of normal hAFCs hybridized with two panel probes. A total of 35-55 nuclei were analyzed per hAFCs culture. Applied panel probes include X-13/18/21 (Red/Green/Blue/Orange, respectively) and Y-15/16/22 (Red/Green/Blue/Orange, respectively).

Generally, the statistical analysis of the results demonstrated that the frequency of polyploidy and total chromosomal abnormalities were significantly different (Table II), so that they increase significantly in DMEM compared to the AmnioMAX group. Our finding shows a large proportion of the total chromosomal abnormalities belongs to polyploidies. The results showed that while in DMEM-cultured AFCs, 5% polyploidy and around 4% other chromosomal abnormalities were observed, in AMX-cultured AFCs, 1.7% polyploidy and around 2.5% other chromosomal abnormalities were recorded. It is worth noting that many of these polyploidy were tetraploid; therefore, they might be normal split signals concerning FISH technique errors (Figures 4 and 5). Interestingly, in most of the samples, which were cultivated for > 10 passages, and one specimen that originally contained trisomy 21, there were no cellular abnormalities due to replicative senescence.

**Table 1:** Rate of aneuploid cells per each chromosome in hAFCs following long-term cultures under two different mediums using the FISH technique and regarding 8 chromosomes


**hAFCs**		**DMEM medium (n = 535)**	**Amnio MAX medium (n = 510)**	**Unpaired ** ***t*** ** test**	
		**95% CI of difference**	**P-value**
**Aneuploidy per chromosome **					
	**13**	0.62 ± 1.62	0	(-0.31, 1.54)	0.18
	**15**	0.82 ± 1.69	0.38 ± 1.39	(-0.82, 1.69)	0.48
	**16**	0.91 ± 2.55	0.52 ± 1.3	(-1.26, 2.02)	0.63
	**18**	0.91± 2.55	0.93 ± 2.28	(-1.98, 1.94)	0.98
	**21**	0.62 ± 1.62	0.08 ± 0.28	(-0.40, 1.48)	0.25
	**22**	0.61 ± 1.60	0.22 ± 0.78	(-0.62, 1.41)	0.43
	**XY**	1.38 ± 2.36	0.52 ± 1.30	(-0.70, 2.40)	0.27
**Total**		4.16 ± 5.93	2.59 ± 3.51	(-2.37, 5.52)	0.42
Data presented as Mean ± SD. Student's *t* test Chr: Chromosome; DMEM: Dulbecco's modified eagle medium; Amnio MAX: AmnioMAX II complete medium					

**Table 2:** Rate of polyploidy, chaotic, and total chromosomal abnormalities in hAFCs following long-term cultures and under two different mediums using the FISH technique and regarding 8 chromosomes


		**Unpaired ** ***t*** ** test**	
**Chromosomal abnormality**	**DMEM medium (n = 535)**	**Amnio MAX medium (n = 510)**	**95% CI of difference**	**P-value**
			**Polyploidy**	5.01 ± 5.22	1.75 ± 2.62	(-0.08, 6.61)	0.056
**Chaotic**	0.99 ± 1.70	0	(0.02, 1.97)	0.04
**Total **	9.57 ± 8.11	4.32 ± 3.33	(0.23, 10.26)	0.04
Data presented as Mean ± SD. Student's *t* test Chr: Chromosome; DMEM: Dulbecco's modified eagle medium; Amnio MAX: AmnioMAX II complete medium				

## 4. Discussion

During the last decade, many authors have pointed out that stem cells barely maintain their chromosome stability throughout long-term expansion (16). In the current study, we aimed to investigate the numerical and structural chromosomal abnormalities of human amniocytes in passages 2-3, as well as polyploidy and aneuploidy of chromosomes 13, 15, 16, 18, 21, 22, X, and Y in these cells when they senesced during passages 3-17. In the clinical diagnosis, such abnormalities raised due to culture are called pseudomosaicism. While there are lots of reports about the pseudomosaicism in AFCs, they are typically restricted to early passages of AFCs to avoid misdiagnosis and make a detailed assessment of PND results. Accordingly, our results revealed a pseudomosaicism but polyploidy with frequency around 1% regarding 650 cases analyzed in primary cultures. However, much more comprehensive studies have recorded further structural and numerical CIN in AFCs. For example, Peakman and colleagues reported 29 cases as pseudomosaicism among 1,100 samples referred for amniocentesis (17). In contradiction to our results, all aberrations were structural [e.g., t (l; 13) and t (9; 14)] and numerical in terms of different aneuploidies, for example, trisomy 2 and 7, without any polyploidy. Hsu and Perlis conducted a nationwide research through 59 cytogenetic laboratories on approximately 60,000 amniocenteses in the United States (18). They reported the average frequency of CIN in single and multiple cells as 2.47% and 0.7%, respectively. Chromosome 2 was most frequently involved in trisomy. Interestingly, a survey of structural abnormalities revealed that there is a significant correlation between the relative chromosome size and structural rearrangements.

In comparison with other human cells, our study evidenced that human amniocytes apparently demonstrate lower CIN compared to other cultured cells. For example, as the chromosomal abnormalities are the hallmark of many tumor cells, the frequency of aneuploidies in tumor cells is quite high, between 5 and 25% of the investigated cells based on cell line. Also, a whole investigation on > 150 hESC lines and 220 iPSC lines have shown that approximately 12-13% of hESCs and iPSCs exhibit chromosome abnormalities, though the majority of the lines were normal in karyotype (19).

Further, the variation in the frequency of chromosomal abnormalities is a major incident involving most stem cell cultures, especially in somatic stem cells in which the aberration ranges from 10-15% of investigated cells in human hematopoietic stem cells and 15-20% in human bone marrow-derived MSCs (hBM-MSCs) (20). Based on numerous reports, adult stem cells establish various chromosomal abnormalities within the 10 passages of in vitro expansion, however, independent laboratories have reported contrasting results in this regard (20). For instance, while Sense be and colleagues revealed that adult MSCs sustain a stable chromosomal integrity throughout long-term cultures (21), Ben- David and coworkers concluded from gene expression profiles that human adult MSC lines contain 4% of chromosomal abnormalities during in vitro expansion (22). However, the proportion and variety of these aberrations vary greatly, depending on the source of adult MSCs as well as laboratories conducting the investigations. For example, Nikitina and colleague declared that hBM-MSCs acquired monosomies of chromosomes 13 and X in almost 1% of analyzed cells (23). Nevertheless, Grigorian and co-workers have demonstrated the trisomy of chromosomes 5, 8, and 20 in 15-20% of analyzed cells in hBM-MSCs (24). Interestingly, some authors recorded such abnormalities in primary few passages, including Estrada and coworkers who reported a trisomy of chromosomes 8 or 17 and a tetrasomy of chromosomes 11 or 17 in 8-12% of analyzed cells during only two passages of human adipose-derived MSCs (25).

Like adult MSCs, comparable discordant results have been obtained for fetal stem cell lines based on the tissue where they originate from, such as umbilical cord blood-derived MSCs (UCB-MSCs), cord blood-derived endothelial progenitor cells (CB-EPCs), placenta-derived MSCs (PL-MSCs), hematopoietic stem cells (HSCs), etc. In this regard, one of the most investigated fetal stem cells is HSCs in which the frequency of CIN varies greatly, depending on the lines reported by independent laboratories. For example, Ben-David and colleagues claimed that CD34${}^{+}$ HSCs isolated from UCB preserve the normal chromosome complement, though without in vitro expansion. Hence, as HSCs are not routinely cultured for multiple passages, the absence of aberrations in their study would not necessarily validate HSCs' resistance to chromosomal abnormality (22). Conversely, another paper revealed that CD34${}^{+}$ UCB-derived HSCs are actually very susceptible to culture-based aberrations since, only during 7-14 days, in vitro conditions established various chromosomal abnormalities in 10-15% of cells, including the tetraploidy of chromosome 13, loss of an X chromosome, and other structural and numerical aberrations especially in chromosome 8, that is, 46,XX,t (2; 8) and 45,XX,-8. It seems that the consequence of culture in CB-EPCs is much worse than UCB-HSCs. Accordingly, Corselli and colleague have shown that among seven CB samples, only two cases preserved normal karyotype throughout 10 passages (< 30%). The rest of the samples presented tetraploidy (92,XXYY; 33% of the cells at passage 2) and a variety of aneuploidies even after two passages, such as +77,XX (68% of cells) and +55,XX (100% of cells) (26).

Noticeably, around 25% of the investigated lines in our study were totally normal (precisely 30% and 23% of samples in AmnioMAX and DMEM, respectively). Alternatively, abnormal samples express a very low level of CIN in terms of total chromosomal abnormality, < 5% and 10% of the analyzed cells in the AmnioMAX and DMEM, respectively. Therefore, compared to other fetal cells, AFCs exhibit a higher stability in genome integrity, both in extremely proliferating and senescent situations. However, aging is definitely correlated with the occurrence of chromosome mis-segregation and consequently chromosomal abnormalities due to general defects in the process of mitosis (27). There are numerous mechanisms underlying CIN, such as defects in cell-cycle regulation, centrosome copy number and mitotic checkpoint function, failing in chromosome cohesion, and kinetochore-microtubule interaction.

Like other human cells, AFCs are able to propagate only in a limited cell divisions under standard culture conditions, a process called replicative senescence. Sustained cell cycle traverse is one of the most famous triggers of cellular senescence induced by telomere attrition (28). Recently, many authors have revealed that human stem cells suffer from a high proportion of chromosomal abnormalities during long-term cultures (19) that hinder their therapeutic potential. It is really hard to come up with a definitive answer to the question why the rate of aneuploidy is low in senesced AFCs. Over the past decades, numerous studies have shown a well-documented link between aging and aneuploidy (27). These studies have reported a positive correlation between the incidence of chromosome mis-segregation and the increase of age-dependent aneuploidies (12, 19, 29). This can raise the question whether senesced cells have a major defect in their mitotic apparatus. However, the notion seems debatable in AFCs, because despite the low rate of aneuploidy in senesced AFCs, they are arrested due to senescence. This could be as a result of simultaneous decrease of telomere length and telomerase activity in senesced AFCs. Mosquera and colleague reported for the first time the telomerase activity in human amniocytes (30). Besides human amniocytes, telomerase activity is found in most immortalized cell lines, such as hESCs, human germ cells, and 80-90% of human tumor samples (31). Sequential sub-culture of human AFCs leads to replicative senescence. As cells enter the terminally non-dividing state, their morphology changes and telomere length progressively decreases during the DNA replication. It seems the decreased telomerase activity in senesced AFCs could be related to a progressive reduction in the subpopulations with higher levels of telomerase activity (30).

Besides calculating the CIN frequency in AFCs, the other aim of our research was to determine the possible effects of culture medium on chromosome aberrations. In this regard, our results indicate that the rate of total aneuploidy and polyploidy as well as total chromosomal abnormalities are significantly higher in the DMEM group. Some former studies have proved that culture conditions can alter the CIN frequency in AFCs. For instance, Bartnitzke and colleague (32) demonstrated that the use of Chang medium elevates the structural CIN of chromosome 1 in periheterochromatic areas so that the rate of abnormality in AFCs cultivated using the Chang medium and medium 199 supplemented with Earle's salts and 20% FBS was 3.7% and 1.4%, respectively. Similar results concerning the effects of Chang medium on chromosome 1 have been reported by Bui and colleague (33). Likewise, Krawczun *et al*. investigated CIN on 212 cases cultured in both the Chang and RPMI-1640 mediums (34). They found a significant correlation between the frequency of CIN and the mediums used to culture the AFCs. About 18 out of 212 amniotic fluid specimens had apparent pseudomosaicism, so that 1.32% of the analyzed cells (scored from 4,908 total cells) suffered from CIN, of which 52.2% were from the Chang medium cultures and 47.8% from RPMI-1640 cultures. Similar studies demonstrated that MSCs derived from different origin could be expanded in a xeno-free supplement for cell culture (SCC) derived from human plasma, without any significant genetic instability and with multipotentiality and distinctive hMSCs phenotype (35). Moreover, a number of studies report that in addition to media formulations, using different enzymatic or non-enzymatic passaging methods may affect the genetic or epigenetic stability of hMSCs. These findings suggest that it will be important to minimize such phenotypic, genetic and epigenetic instabilities in hMSCs cultures especially in clinical applications (36).

## 5. Conclusion

The explanation of CIN and senescence of cultured MSCs is controversial. Previous findings in the literature, as our results, have indicated that elder cells divide slower until when cells go through senescence. The majority of chromosome alterations will be spontaneously eradicated from the culture within a few passages, whereas the senescent cells retain their aberrations because they cease to divide. Despite the fact that a number of reports have outlined the role of aneuploidy as a key trigger of senescence phenotypes, in our study, as mentioned earlier, 8 and 6 out of 13 cases in AmnioMAX and DMEM groups, respectively, showed no evidence of aneuploidy. Of note, due to the limited access to modern technologies, structural abnormalities have not been considered and more thorough investigations involving all chromosomes are recommended. Taken together, our finding indicates the chromosomal stability of hAFCs under different culture conditions and in both early and long-term cultures. Therefore, they could be considered as an attractive cell source for the derivation of stem cells with therapeutic potentials in regenerative medicine.

##  Conflict of Interest 

The authors declare that there is no conflict of interest regarding the publication of this article.
